# Self-Reported Gastrointestinal Symptoms Two To Four Years After Bariatric Surgery. A Cross-Sectional Study Comparing Roux-en-Y Gastric Bypass and Laparoscopic Sleeve Gastrectomy

**DOI:** 10.1007/s11695-021-05605-5

**Published:** 2021-08-10

**Authors:** Brit Thorsen, Kari Hanne Gjeilo, Jorunn Sandvik, Turid Follestad, Hallvard Græslie, Siren Nymo

**Affiliations:** 1 Nord-Trøndelag Hospital Trust, Clinic of Surgery, Namsos Hospital, Namsos, Norway; 2 Department of Circulation and Medical Imaging, Faculty of Medicine and Health Sciences, Norwegian University of Science and Technology, Trondheim, Norway; 3 Department of Cardiology, St. Olavs Hospital, Trondheim University Hospital, Trondheim, Norway; 4 Department of Public Health and Nursing, Faculty of Medicine and Health Sciences, Norwegian University of Science and Technology, Trondheim, Norway; 5 Department of Surgery, Møre and Romsdal Hospital Trust Ålesund, Ålesund, Norway; 6 Department of Clinical and Molecular Medicine, Faculty of Medicine and Health Sciences, Norwegian University of Science and Technology, Trondheim, Norway; 7 Center for Obesity, Department of Surgery, St. Olav Hospital, Trondheim University Hospital, Trondheim, Norway

**Keywords:** Pain, Reflux, Bariatric surgery, Gastrointestinal symptoms, PROMs

## Abstract

**Background:**

Roux-en-Y gastric bypass (RYGBP) and laparoscopic sleeve gastrectomy (LSG) are efficient methods for weight loss (WL) and WL maintenance in severe obesity. However, the knowledge of gastrointestinal (GI) symptoms after surgery is limited. This study aimed to compare the severity of GI symptoms, pain, and self-rated health 2 to 4 years after RYGBP and LSG surgery.

**Methods:**

In this cross-sectional study, RYGBP and LSG patients answered a questionnaire including the Gastrointestinal Symptom Rating Scale (GSRS), questions from the Brief Pain Inventory (BPI), and self-rated health (SRH).

**Results:**

A total of 172/303 (57%) responded, RYGBP (*n*=73) and LSG (*n*=99). The mean age was 45.3 (SD 11.1) years (74% females). There was no evidence of a difference in total GSRS scores between the surgical methods (*p*=0.638). There were higher scores of reflux symptoms in LSG vs. RYGBP (both median 1, 75-percentile 2.5 vs. 1.0, *p* <0.001) and higher consumption of acid-reducing medication after LSG (32% vs. 12%, *p* <0.001). Pain scores were low in both groups; however, average abdominal pain was higher for RYGBP, median 2 (IQR 0–4) vs. median 1 (IQR 0–3) for LSG (*p* = 0.025). There was no significant difference in SRH.

**Conclusions:**

Patients undergoing RYGBP and LSG surgery reported similar total GSRS scores and low pain scores 2 to 4 years after surgery. However, reflux symptoms and use of acid-reducing medication occurred more frequently after LSG surgery, while abdominal pain was more frequent in RYGBP surgery. These findings are important for surgical decision-making and follow-up.

**Graphical abstract:**

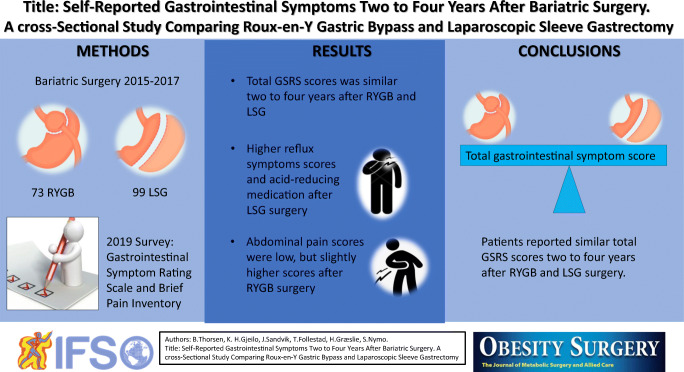

## Introduction

Roux-en-Y gastric bypass (RYGBP) and laparoscopic sleeve gastrectomy (LSG) are the most common bariatric surgeries worldwide [1]. Both procedures are efficient for weight loss (WL) and WL maintenance and for remission of comorbidities such as type 2 diabetes mellitus (T2DM) and cardiovascular diseases [2]. The risk of surgical complications, nutritional deficiency, and gastrointestinal (GI) symptoms are important considerations for treatment choices. However, self-reported GI side effects are generally poorly studied [3]. LSG is known to give more reflux symptoms than RYGBP [4] and also to cause de novo reflux symptoms after surgery [5]. Surgical technique, older age, smoking habits, comorbidities, % excess weight loss (EWL), and eating behavior are factors associated with postoperative reflux [6, 7]. RYGBP is associated with development of recurrent or chronic abdominal pain in 30–43% of patients in the long term [8, 9]. Contact with the healthcare system due to abdominal pain is reported more frequently after RYGBP compared to obesity in general [10, 11]. There is not one consistent cause of pain after RYGBP, but dumping syndrome, bowel obstruction, gallstone-related disease, anastomotic ulcers, dysfunctional eating, and food intolerance are possible explanations [12–16]. Abdominal pain after RYGBP is more common in younger women and associated with lower education levels, smoking, and total bodily pain [10, 17]. Both RYGBP and LSG impose physiological and anatomical alterations that may cause GI symptoms, but GI symptoms may be related to a variety of physical and psychological conditions. Self-rated health (SRH) is a good predictor of morbidity, mortality, and healthcare use [18], and chronic pain is independently associated with SRH [19]. Most patients experience improvement of quality of life (QoL) after RYGBP or LSG surgery [20, 21], often related to WL [22, 23] but also to reduced prevalence and intensity of GI symptoms and abdominal pain [9, 10].

To ensure the best possible outcome and the fewer complications and symptoms after surgery, the choice of either RYGBP or LSG treatment is important both from a patient and socioeconomic perspective. The knowledge of bariatric surgery and GI symptoms beyond 1 or 1 years is limited, especially after recommendation for routine closure of mesenteric defects for RYGBP. Further, studies of both abdominal pain and reflux symptoms are scarce. This lack of knowledge is a challenge for treatment choices where patient-centered approaches including patient information and shared decision-making are fundamental. Therefore, the aim of this study was to compare self-reported GI symptoms and SRH between RYGBP and LSG 2 to 4 years after surgery.

## Material and Methods

All patients aged 18 to 66 years, who underwent primary RYGBP or LSG between 2015 and 2017 at a local hospital in Norway, were invited to answer a questionnaire in a cross-sectional study in 2019. Previous bariatric surgery and unknown private addresses were exclusion criteria. The Regional Committee for Medical and Health Research Ethics approved the study (REK 2019/51, Central-Norway).

### Surgical Technique

RYGBP involved laparoscopic formation of a 30 ml gastric pouch with a 100–150 cm alimentary limb, a 40–60 biliopancreatic limb, and closure of the mesenteric defects. A 32-Fr bougie was used for calibration in all LSG surgeries. All patients underwent routine preoperative gastroscopy and triple therapies for *Helicobacter pylori* (HP) eradication if HP infection.

### Data Collection

The questionnaire was sent by mail, and the participants completed a study-specific questionnaire, the Gastrointestinal Symptom Rating Scale (GSRS), questions from the Brief Pain Inventory (BPI), and self-rated health (SRH).

The study-specific questionnaire included self-reported sociodemographic and clinical information. The patients were also asked whether, in retrospect, they regretted the surgical procedure.

GSRS is a validated questionnaire consisting of 15 gastrointestinal symptom items, scored on a 7-point Likert scale (1= no discomfort and 7= severe discomfort) combined into the following clusters: abdominal pain, reflux, diarrhea, constipation, and indigestion [24, 25]. A cluster score was calculated only when all items in the cluster were answered. The means of the total score and of the scores for each symptom cluster are presented. GSRS has also been used in previous studies of bariatric surgery [9, 14, 17, 26–30].

BPI is a validated questionnaire frequently used to assess pain in different surgical populations [31, 32], bariatric surgery included [17]. In this study, we used two modified questions to assess severity of pain: worst abdominal pain in the last 24 h and abdominal pain on average (without time frame), rated on an 11-point numeric rating scale (NRS) from 0 (no pain) to 10 (worst imaginable pain).

Self-rated health (SRH) is a simple, spontaneous subjective assessment of a person’s health status rated on a 5-point scale from excellent to poor [18]. SRH is a relevant and valid outcome measure for bariatric surgery [20].

The Anatomical Therapeutic Chemical (ATC) Classification System was used to classify self-reported acid-reducing medication, proton pump inhibitors (ATC A02B), and histamine H2-receptor antagonist (ATC A02B A).

### Statistical Analysis

Data are summarized using means (SD), medians (25- and 75-percentiles), or frequencies (%) as appropriate. The *t*-test, Mann-Whitney *U*-test, chi-square test, or Fischer’s Exact test was used to compare continuous, ordinal, or binary variables between the surgery types as appropriate. In addition, a multiple linear regression was used to study the association between the natural logarithm of total GSRS score and the explanatory variables surgical method, gender, age, % total weight loss (TWL), smoking habits, and time after surgery (months). All analyses were performed with SPSS version 25 (SPSS IBM, New York, USA). Statistical significance was assumed at *p*<0.05.

## Results

In total, 323 patients underwent RYGBP (*n*=153) or LSG (*n*=170) surgery between January 2015 and December 2017. Six percent (*n*=15) were excluded due to unknown private addresses (RYGBP 3, LSG 5), due to being deceased at the time of the survey (RYGBP 2) and previous bariatric surgery (RYGBP 5). The response rate was 57% (*n*=172). There was no statistically significant difference in response rate between the two types of bariatric surgery (RYGB 51% vs. LSG 62%, *p*=0.064). The patients responded between 17 and 52 months after surgery (mean 33.0 (SD 10) months). Participant characteristics are presented in Table 1. The mean age was 45.3 (11.1) years, and 74% were women. There was no statistically significant difference in marital status, education, working situation, or smoking habits between the RYGBP group and the LSG group. There was a higher %TWL after RYGBP: 36.4% (7.5) vs. 30.4% (9.5) for LSG (95% CI for difference 3.4–8.7% points, *p* <0.001).

**Table 1:** Participant characteristics

	Total		RYGBP		LSG		*p*-value
	*n*=172		*n*=73		*n*=99		
Sex, *n* (%)							0.972
Female	127	(73.8)	54	(74.0)	73	(73.7)	
Male	45	(26.2)	19	(26.0)	26	(26.3)	
Age (years), mean (SD)	45.3	(11.1)	43.8	(10.7)	46.3	(11.1)	0.147^a^
BMI (kg/m^2^) preop, mean (SD)	44.4	(6.0)	43.6	(4.5)	45.0	(6.8)	0.110^a^
Marital status, *n* (%)							0.185
Married	67	(39.0)	26	(35.6)	41	(41.4)	
Cohabited	52	(30.2)	29	(39.7)	23	(23.2)	
Single	37	(21.5)	13	(17.8)	24	(24.2)	
Widowed	1	(0.6)	0	(0.0)	1	(1.0)	
Divorced	15	(8.7)	5	(6.8)	10	(10.1)	
Highest educational level, n (%)							0.811
Primary school	21	(12.2)	8	(11.0)	13	(13.1)	
3 years high school	40	(23.3)	15	(20.5)	25	(25.3)	
Certificate of apprenticeship	49	(28.5)	23	(31.5)	26	(26.3)	
College/university, 1-3 years	38	(22.1)	18	(24.7)	20	(20.2)	
College/university, 4 years or more	24	(14.0)	9	(12.3)	15	(15.2)	
Working / studying today, n (%)							0.197
Work/study 80% or more	95	(55.2)	46	(63.0)	49	(49.5)	
Work/study 20-80%	25	(14.5)	8	(11.0)	17	(17.2)	
No work or study	52	(30.2)	19	(26.0)	33	(33.3)	
Smoking, n (%)							0.762
Never smoked	137	(79.7)	57	(78.1)	80	(80.8)	
Occasional	20	(11.6)	10	(13.7)	10	(10.1)	
Daily	15	(8.7)	6	(8.2)	9	(9.1)	
BMI at servery, mean (SD)	29.6	(5.3)	27.5	(3.7)	31.1	(5.7)	**<0.001**^a^
%TWL, mean (SD)	32.8	(9.2)	36.4	(7.5)	30.4	(9.5)	**<0.001**^a^
%EWL, mean (SD)	77.8	(20.9)	87.4	(18.0)	70.7	(20.1)	**<0.001**^a^

Results are presented as mean (SD) and categorical variables as frequency (%)

^a^*p*-value for a *t*-test, otherwise for a chi-square test, for difference between RYGBP and LSG

*RYGBP* Roux-en-Y gastric bypass, *LSG* laparoscopic sleeve gastrectomy, *BMI* body mass index, *kg/m*^*2*^ kilogram per square meter, *TWL* total weight loss, *EWL* excess weight loss

Missing height and weight for 1 LSG

**Fig. 1:**
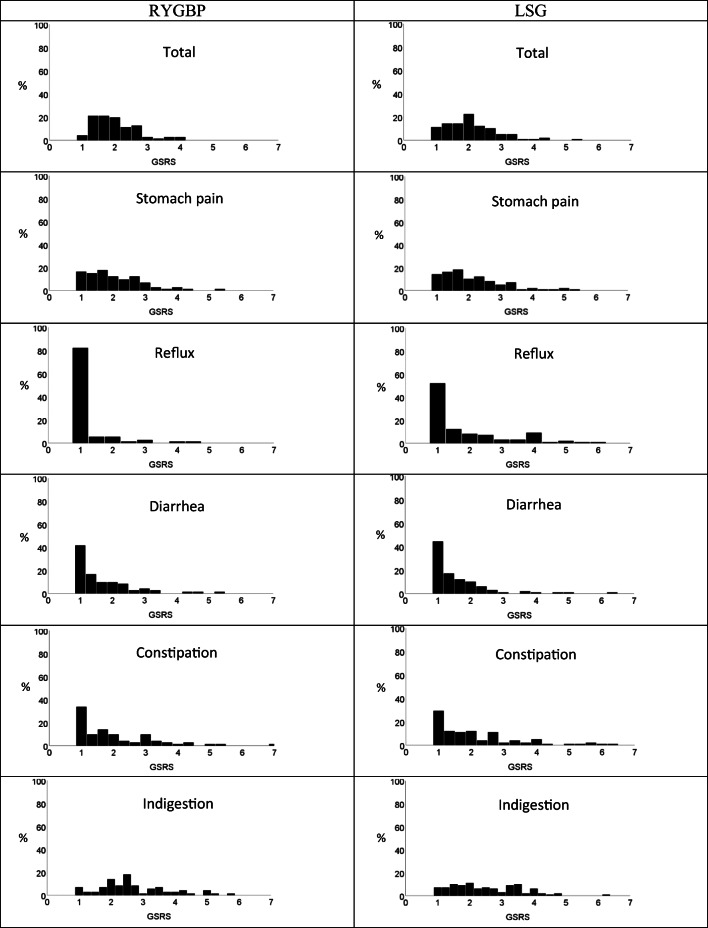
Intensity of gastrointestinal symptoms 2 to 4 years after Roux-en-Y gastric bypass (RYGBP) and laparoscopic sleeve gastrectomy (LSG). Total score on average on top row, for each cluster below. Gastrointestinal Symptom Rating Scale (GSRS) score 1–7 (1= no discomfort and 7= severe discomfort).

The GSRS scores 2 to 4 years after surgery are presented in Table 2 and Figure 1. There was no evidence of a difference in total GSRS scores between RYGBP and LSG, or in the domains abdominal pain, diarrhea, constipation, and indigestion. The GSRS reflux score was significantly higher for LSG than for RYGBP (median 1.0 (1.0–2.5) vs. median 1.0 (1.0–1.0), *p* <0.001). Use of acid-reducing medication was higher for LSG (32% vs. 12%, *p*<0.001). Results from the multiple linear regression analysis are presented in Table 3. After adjusting for gender, age, %TWL, smoking habits, and number of months after surgery, there was no significant difference (on a log-transformed scale) in total GSRS scores between the surgical methods; however, the estimated difference between smokers and non-smokers was 0.167 (95% CI 0.035–0.299 *p*=0.014). No significant difference for the other factors was found.

**Table 2:** Gastrointestinal symptoms 2–4 years after bariatric surgery, Roux-en-Y gastric bypass, and laparoscopic sleeve gastrectomy

GSRS	Total		RYGBP		LSG		*p*-value
	*n*=172		*n*=73		*n*=99		
	Mean	Median (25-75-perc.)	Mean	Median (25-75-perc.)	Mean	Median (25-75-perc.)	
Total	2.1	1.9 (1.5–2.5)	2.0	1.9 (1.5–2.5)	2.1	2.0 (1.5–2.5)	0.638
Abdominal pain	2.1	2.0 (1.3–2.7)	2.0	2.0 (1.3–2.7)	2.1	2.0 (1.3–2.7)	0.724
Reflux	1.6	1.0 (1.0–2.0)	1.2	1.0 (1.0–1.0)	1.9	1.0 (1.0–2.5)	**<0.001**
Diarrhea	1.6	1.3 (1.0–2.0)	1.6	1.3 (1.0–2.0)	1.6	1.3 (1.0–2.0)	0.529
Constipation	2.1	1.7 (1.0–2.7)	2.0	1.7 (1.0–3.0)	2.1	1.7 (1.0–2.7)	0.600
Indigestion	2.6	2.5 (1.8–3.4)	2.7	2.5 (2.0–3.3)	2.5	2.3 (1.7–3.3)	0.281

Results are presented as mean and median (25- and 75-percentile) of average score

*p*-value for Mann-Whitney *U*-test

*GSRS* Gastrointestinal Symptom Rating Scale, *RYGBP* Roux-en-Y gastric bypass, *LSG* laparoscopic sleeve gastrectomy. Score 1–7 (1= no discomfort and 7= severe discomfort). Missing data for three participants for total score, two for constipation and one for the other domains

**Table 3:** Multiple linear regression with the natural logarithm of total GSRS score as dependent variable

	Estimate coefficient	95% confidence interval	*p*-value
Surgical method (LSG vs. RYGBP)	.012	−.104 to .127	0.841
Sex (women vs. men)	.101	−.020 to .222	0.102
Age	−.003	−.008 to .002	0.279
%TWL	−.003	−.010 to .003	0.326
Smoking (daily/occasional vs. non-smoking)	.167	.034 to .299	**0.014**
Month after surgery	−.003	−.006 to .004	0.706

*GSRS* Gastrointestinal Symptom Rating Scale, *RYGBP* Roux-en-Y gastric bypass, *LSG* laparoscopic sleeve gastrectomy, *TWL* total weight loss

**Fig. 2:**
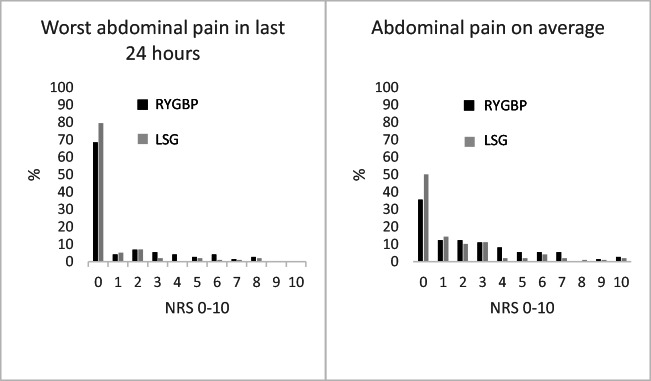
Worst abdominal pain in last 24 h and average abdominal pain 2 to 4 years after Roux-en-Y gastric bypass (RYGBP) and laparoscopic sleeve gastrectomy (LSG). Numeric rating scale (NRS) 0 = no pain at all and 10 = worst imaginable pain.

Pain scores for abdominal pain 2 to 4 years after RYGBP and LSG surgery are presented in Figure 2. Both groups had low scores for abdominal pain. There was slightly higher evidence for a difference in average abdominal pain (*p*=0.025) than in strongest abdominal pain during the last 24 h (*p*=0.067) between the two groups, with highest pain for RYGBP.

**Fig. 3:**
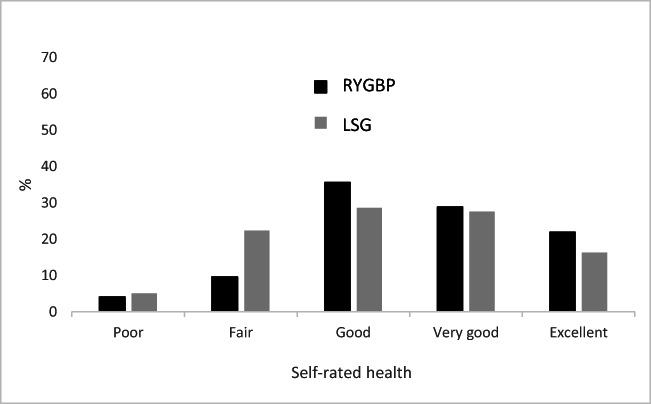
Self-rated health for Roux-en-Y gastric bypass (RYGBP) and laparoscopic sleeve gastrectomy (LSG) patients 2 to 4 years after surgery.

The results for SRH are presented in Figure 3. There was no evidence of a difference in SRH between RYGBP and LSG surgery (*p* = 0.116).

The majority of the participants in either group did not regret having bariatric surgery, 89% RYGBP vs. 94% LSG (*p*=0.343).

## Discussion

This study showed no significant difference in the occurrence of self-reported GI symptoms measured with total GSRS between participants undergone RYGBP and LSG 2 to 4 years after surgery. However, an estimated difference in total GSRS between smokers and non-smokers was found and was highest for smokers. There was a larger occurrence of reflux symptoms and use of acid-reducing medication among individuals after LSG surgery. Individuals who underwent RYGBP surgery had slightly higher scores for average abdominal pain.

The participants reported a slightly lower total median GSRS score for both RYGBP and LSG surgery compared to findings in other studies 2 years after RYGBP surgery [14, 26]. Short-term follow-up studies found decreased total GSRS scores 1 year after both RYGBP and LSG surgery [27, 29] but increased total GSRS scores 2 and 5 years after RYGBP surgery [26, 33]. In this study, no evidence of a correlation between total GSRS scores time after surgery was found. Smoking is a well-known risk factor for postoperative morbidity after bariatric surgery [34], and patients are strictly strongly urged to quit smoking before undergoing bariatric surgery. After RYGBP, smoking is one risk factor for marginal ulceration [15], and generally smoking is a risk factor for both gastroesophageal reflux symptoms [35] and peptic ulcer [36]. The most frequent symptom in both groups was indigestion. The intensity of the symptoms was in line with results from other bariatric surgery studies of symptoms assessed by GSRS [9, 14, 17, 26, 28, 29]. Indigestion is also the most common GI symptom before bariatric surgery [14, 26, 27], but it is rarely a reason to consult the healthcare system after bariatric surgery [10].

Participants reported the second highest score for abdominal pain with 16% of RYGBP and 20% of LSG patients reporting GSRS scores of ≥3. The degree of abdominal pain was in line with other studies with similar surgical procedures [14, 26, 28]. Contradictory, in studies with 5 and 10 years follow-up after RYGBP, 33% and 43% reported GSRS scores of ≥3 for abdominal pain, respectively [9, 17]. Further, in a review by Mala and Hogestol (2018), up to 30% reported abdominal pain after RYGBP surgery [8]. The anatomical changes after RYGBP present a greater risk of ulcer by gastrojejunal anastomosis [15], anastomosis stenosis [37], internal herniation [16], and dumping syndrome [12]. Internal hernia may explain some of the high occurrence of abdominal pain after RYGBP in studies before recommendation for routine closure of mesenteric defects [17]; an occurrence of 7% before and 2% after has been reported [16].

In this study, abdominal pain was also assessed by the NRS 0–10 scale. Abdominal pain ≤3 is considered low and was found in most of the participants. The difference between the surgical methods was small and not considered clinically relevant [38]. Overall, both groups reported higher scores on average than for the last 24 h. Hogestol et al. (2017) found that only 20% of the participants experienced abdominal pain daily, with 50% of these experiencing an intensity >7 (NRS 0–10) [17]. This demonstrates that the intensity of abdominal pains may vary over time. Mala and Hogestol (2018) concluded that the prevalence of abdominal pain after bariatric surgery is uncertain due to the lack of longitudinal studies with preoperative measures of pain [8]. A 2-year follow-up after RYGBP found that patients with preoperative chronic abdominal pain also had higher GSRS scores for abdominal pain 2 years after surgery [30]. Although there is a relatively low incidence of abdominal pain and only a few patients report high intensity pain in this study, it is a problem for those concerned and for healthcare resources [10, 11].

Reflux was more common in the LSG group than in the RYGBP group. Obesity increases the risk of reflux symptoms [35, 39], and usually, RYGBP is found to improve reflux symptoms [33, 40]. A meta-analysis found a higher risk of gastroesophageal reflux disease (GERD) after LSG compared to RYGBP [4] which is in line with this study’s findings. Several studies have found increased or new onset of reflux symptoms in participants undergoing LSG [5, 29, 41]. Peterli et al. (2018) found that 32% in the LSG group had increased reflux symptoms and 25% had remission of reflux [40]. These findings are considered clinically important for deciding whether RYGBP or LSG is best for both those with and without preoperative reflux symptoms. Concern regarding the patients’ ability to adapt to necessary changes in eating and drinking habits after bariatric surgery is one of two main factors leading to self-removal from the bariatric surgery program preoperatively [42]. Loss of control of eating and late evening meals occur after bariatric surgery [7], and eating behavior affects GI symptoms. Smoking is a main risk factor for GERD symptoms [35], and patients’ smoking habits are known to be underreported [43]. In this study, bougie size 32 Fr was used; however, a 36-Fr bougie size was common at this time [44]. According to the literature, there seems to be a relationship between WL and the size of the bougie, but the relationship between reflux and bougie size does not seem clear and could be multifactorial [45]. In a recent study, older patients, smokers, patients with comorbidities, and patients with more EWL had significantly worse GERD symptoms with LSG postoperatively [6].

Use of acid-reducing medication was more common after LSG compared to RYGBP surgery, though the reason for its use was not described by the patients. Although GERD can be relieved by medical treatment, it increases the risk of esophageal cancer [46]. Approximately, up to 70% have silent GERD; therefore the numbers of patients needing treatment is underdiagnosed [47], which is a challenge both at the individual and socioeconomic level.

We found no evidence of a difference in SRH 2 to 4 years after bariatric surgery between the groups, and most of the participants (87 and 71% for RYGBP and LSG, respectively) rated their health as good. Similarly, in a 5-year follow-up study after RYGBP, Sandvik et al. (2019) found improved SRH in two-thirds of the participants [20]. Several studies of bariatric surgery have used more comprehensive patient reported outcome measures (PROMs). Weight loss after bariatric surgery is strongly associated with improvement in QoL [22, 23]. However several other studies have found a negative association between GI symptoms and QoL [9, 10, 28]. Felchenreich et al. (2019) found that reflux was more strongly correlated with self-perceived health than with %EWL 10 years after LSG [48]. Similarly, Biter et al. (2017) found that GERD made the only significant difference in QoL between those operated by RYGBP and LSG [49]. SRH is one of the predictors of changes in employment impairment post-bariatric surgery [50]. Hence, the highest possible SRH score and fewest possible GI symptoms after surgery are important both from a patient and socioeconomic perspective.

There is a lack of valid disease-specific PROMs for bariatric surgery. GSRS does not include all symptoms for dumping syndrome, e.g., sweating, dizziness, and increased heartrate. These are not directly gastrointestinal symptoms, but might be the worst problems related to food intake after RYGB. Both GSRS and BPI only capture physical domains of QoL. The systematic review by deVries et al. (2018) found no QoL instrument specifically recommended for bariatric surgery [51]. However, the BODY-Q was recommended for future research. But, with few available translations and a large number of items (138 items), it is a challenge to implement it in clinical practice and research. Therefore, methodologically validated and disease-specific tools for bariatric surgery are needed.

## Strengths and Limitations

The present study has several strengths. First, there were few exclusion criteria. Second, only one written reminder was sent; volunteering in relation to participation was a vital ethical aspect but may have biased the response rate. Third, both pain and other GI symptoms are subjective symptoms; self-reporting is the gold standard for symptom assessment. GSRS has been used in several studies for bariatric surgery, and the results are comparable.

Fourth, the study had a relatively small sample size, however, comparable with previous studies [9, 17, 26, 30]. More than one and/or different reminder approaches might have increased the response rate and strengthened the study.

Another limitation of the study is that only postoperative data was collected. Further longitudinal studies with preoperative scores and use of multiple valid mapping tools like body chart and eating behavior would provide a better understanding of the association between surgical methods and GI symptoms.

## Conclusions

Patients undergoing RYGBP and LSG surgery reported similar GI symptoms scores 2 to 4 years after surgery. Very few regretted undergoing bariatric surgery, and no differences between groups were found. However, reflux symptoms and use of acid-reducing medication were more frequent after LSG surgery, and an indication of higher, but still moderate, abdominal pain after RYGBP surgery was found. SRH was good independently of surgical method. These findings may be useful in the decision-making process of surgical methods and for personalized lifetime follow-up after bariatric surgery. Furthermore, larger studies with preoperative data, longitudinal designs, and PROMs specific for bariatric surgery are needed to confirm these findings.
